# The impact of income level on skeletal muscle health in rural Chinese older residents: a study of mediating effects based on dietary knowledge

**DOI:** 10.3389/fpubh.2024.1329234

**Published:** 2024-02-14

**Authors:** Xiaochen Zhang, Gangyi Wang, Jiwei Ma, Huijing Bai

**Affiliations:** ^1^School of Management, Harbin University of Commerce, Harbin, China; ^2^School of Economics and Management, Northeast Agricultural University, Harbin, China; ^3^Nutrition Department, Huadong Hospital Affiliated to Fudan University, Shanghai, China

**Keywords:** income level, rural older residents, skeletal muscle health, sarcopenia, dietary knowledge

## Abstract

China’s rural residents have basically solved the problem of subsistence, but due to aging, the prevalence of sarcopenia (abbreviated as sarcopenia) has been increasing year by year, especially the skeletal muscle health of the rural older residents has not been sufficiently paid attention to, so analyses of the impact of income level on the skeletal muscle health of the older people in rural areas of China are of great practical significance. Based on the annual data of the China Health and Nutrition Survey (CHNS) in 2006, 2009, and 2011, we introduced the mediator variable of dietary knowledge and used the Probit model regression, mediation effect model, and instrumental variable regression to assess the skeletal muscle health status of the rural older people in China and explore the mechanism of the influence of the income level on the skeletal muscle health of the rural older residents in China. The primary objectives of this study were to evaluate the impact of income level on the skeletal muscle health status of older adults living in rural areas of China and to investigate the underlying mechanisms. By analyzing the findings of this study, our aim is to establish a correlation between the economic status and skeletal muscle health of older adults in rural communities, as well as elucidate the influence of income level and dietary knowledge on their skeletal muscle health. Through the attainment of these objectives, we hope to provide valuable insights and recommendations for enhancing skeletal muscle health among the rural older population in China. Based on our research findings, it can be inferred that there was a significant association between the financial status of rural older adults and their skeletal muscle health. Additionally, the prevalence of sarcopenia was lower among individuals with higher income levels, and there was a negative correlation between the prevalence of sarcopenia and the level of dietary knowledge among rural older individuals. The knowledge of dietary knowledge level of rural older people plays a mediating role in the income level and the prevalence of sarcopenia. Moreover, with the change in income level and the increase in age, the change in skeletal muscle health status showed obvious heterogeneity, in which the effect on the relatively younger (65–70 years old) samples was greater. Therefore, sustained income growth remains an effective way to improve the skeletal muscle health of older rural residents. At the same time, improving dietary knowledge and dietary quality among the older people is important in preventing a decline in muscle strength and physical function and in preventing the onset of sarcopenia.

## 1 Introduction

The concept of Sarcopenia was first coined by Irwin Rosenberg, a professor at Tufts University in the United States of America, to mean muscle loss (1). The most common causes of sarcopenia are aging, reduced activity, disease, and malnutrition. Sarcopenia can be divided into two main categories: primary and secondary, primary sarcopenia is mainly associated with aging (2). If other comorbidities are present along with aging, the condition is known as secondary sarcopenia. The condition known as secondary sarcopenia is more common than primary sarcopenia and requires special attention (3). According to the latest World Population Ageing Report of the United Nations Economic and Social Office, population aging has become a global trend. The Manpower Planning Office (MPO) predicts that China’s population over 65 years old will grow from 10.7% in 2010 to more than 25% in 2025, and even to 41.6% in 2065 (4). According to the latest data from the National Bureau of Statistics (NBS), China’s aged 65 and above is as high as 158.31 million, accounting for 11.4% of the total population. When the older population aged 65 and above accounts for 7% of the total population in a country or region, it means that the country or region is in an aging society (5). Therefore, China has entered an aging society. With the increase of the aging population, the physiological condition of the older people will deteriorate, their physical mobility will decrease, and the society of chronic diseases will increase, further accelerating the attenuation of skeletal muscle, and leading to sarcopenia. Therefore, the prevention of sarcopenia has become even more essential, and the older population is the main focus of scholars’ research on the problem. As a result, preventing sarcopenia has become increasingly crucial, with scholars primarily focusing their research on the older population. Our objective is to offer valuable insights and recommendations aimed at improving skeletal muscle health among rural older adults in China. By doing so, we aspire to contribute to the overall well-being and quality of life of this demographic.

The implementation of the rural revitalization strategy was a major strategic decision made by the 19th Party and is the general gripping force of the work of “three rural areas” in the new era of socialism with Chinese characteristics. Currently, we are in the hard and decisive period of poverty alleviation, the start of the implementation of the rural revitalization strategy, and the intersection of poverty alleviation and rural revitalization (6). There are also many international studies on the relationship between income and sarcopenia. For example, Daskalopoulou Christina et al. suggested in their study that there may be a significant association between gender, marital status, education, personal economic status, and the chance of developing sarcopenia (7). Ahmadreza Dorosty suggested that there is a highly significant correlation between socioeconomic status and sarcopenia (*p* < 0.001) and that people with low socio-economic status people have 0.97 times the risk of developing sarcopenia than those in the middle and high-income brackets (8). Cassie Jeng stated that Asians have the highest prevalence of sarcopenia and black people have the lowest groups. Income level and education level both affect the prevalence of sarcopenia to a great extent in both males and females (9). Scientific studies have also shown that people of lower economic levels are more likely to overspend on healthcare and experience greater financial stress than the relatively affluent. The prevalence of sarcopenia further increases the risk of catastrophic health expenditure in the lowest socio-economic groups (10). The available evidence suggests that income is an important determinant of sarcopenia risk in older adults, but further detailed studies are needed on socioeconomic-specific pathways through which income influences sarcopenia and to make recommendations to improve skeletal muscle health in older adults.

With the rapid development of China’s social and economic development, the dietary structure and eating habits of Chinese residents have also produced great changes, dietary characteristics gradually tend to be high calorie, high fat, and high sugar patterns, the risk of chronic diseases has also increased dramatically (11). In their study, Zhang Yan and Jin Shaosheng showed that consumers’ personal characteristics, economic factors, cognitive and attitudinal variables, and time effects had a significant impact on the decision-making process of dairy product “consumption participation,” which was mainly manifested in the fact that the higher the level of income, the higher the level of education, and the smaller the BMI value of the urban residents, the higher the degree of understanding of the Dietary Guidelines and the level of their own knowledge of the diet in general (12). According to Kang Houang, there are significant differences in dietary and nutritional knowledge, attitudes, and behaviors between low-income and high-income groups. There are two main reasons for this: firstly, high-income people can generally get better protection for their material life, and they are more capable, and they have some energy to search for channels so as to improve their dietary knowledge; on the other hand, low-income people have a relatively low level of dietary knowledge due to their economic constraints and limited energy (13). Existing evidence suggests that lower socioeconomic status is associated with an increased risk of sarcopenia (14, 15), and factors such as income, education (16, 17), and occupation (18) can have a significant impact on skeletal muscle health in older adults. Li Cheng extracted and analyzed the dietary patterns of older people from three regions by exploratory factor analysis and explored the relationship between different dietary patterns and muscle wasting disease in older people, and further analyzed the relationship between dietary nutrients and macronutrient energy supply ratios and muscle wasting disease in the older people from the perspective of dietary patterns, and explored the possible ways in which dietary patterns affect muscle wasting disease in the older residents and the related mechanisms (19). Researchers abroad have also pointed out that diet is an important exogenous factor in disease, that the development of sarcopenia is closely related to diet, and that healthier dietary patterns can help reduce the risk of sarcopenia (20).

In a study evaluating the impact of a nutrition intervention on promoting healthy eating knowledge and eating practices among adolescents, the researchers suggested that the proposed intervention increased adolescents’ dietary knowledge while improving some of their eating practices. The authors concluded that the use of a problem-posing approach and the use of food illustrations for educational activities were effective in promoting healthy eating practices among adolescents (21). Huan Wang et al. noted that nutrition and dietary education are effective in improving the dietary and nutritional knowledge and practices of people with diabetes and that such best practices help them to effectively control their blood glucose (22). Following this, in a study on the reliability of a dietary questionnaire designed to assess the eating habits, eating behavior, and nutritional knowledge of adolescents, the researchers designed the questionnaire in such a way as to identify nutritional knowledge, knowledge of food safety, etc., as the main factors influencing the eating habits of adolescents and as a basis for the possibilities of improving eating habits (23). Simultaneously, a recent national study of 697 Chinese adolescents (aged 12–17 years) showed that dietary and nutritional knowledge and social attitudes were among the main predictors of food preferences (24). Jane Kolodinsk, in a study of college students’ knowledge of current dietary guidelines and college students’ food choices, concluded that dietary knowledge was associated with making healthier food choices and that increased dietary knowledge was positively correlated with healthier eating patterns. Overall, those with better dietary practices had higher levels of dietary knowledge. The authors suggest that dietary guidelines should be combined with effective public awareness campaigns and thus become an effective mechanism to promote changes in household dietary choices (25).

However, there are no studies that directly show whether people’s level of dietary knowledge can directly affect skeletal muscle health, so how dietary knowledge specifically affects skeletal muscle health still requires further research.

As of now, there are a number of methods available to assess skeletal muscle mass, skeletal muscle strength, and skeletal muscle function, researched fat-free body weight, calf circumference, upper arm circumference, skin fold thickness measurements, grip strength, knee flexion/extension, relative skeletal muscle index, electromyography, gait analysis, lower extremity strength, CT, magnetic resonance imaging (MRI), and ultrasound (26), CT, magnetic resonance imaging (MRI), and ultrasound testing (26). However, there has been a lack of recognized diagnostic criteria for sarcopenia, and Baumgartner RN et al. published a diagnostic method for sarcopenia in 1998, which uses height-related muscle mass to diagnose the degree of sarcopenia (27). Appendicular skeletal muscle mass (ASM) is measured using the DXA, and the ratio of the square of the skeletal muscle mass of the limbs (kg) to the height (m) is the skeletal muscle index (SMI); if the SMI is less than 2 standard deviations below that of a healthy young person of the same sex, then the person is likely to have sarcopenia. Then there is a high probability of having sarcopenia (28). Bioelectrical impedance analysis (BIA) can also be used, which does not measure muscle mass directly but gives an estimate of muscle mass based on whole-body conductivity and is affordable, widely available, and portable (29). In addition to this, measures of physical function such as the balance test, 4 m timed walk test and timed sit-to-stand test can add to the diagnostic strength of sarcopenia, and these tests can predict the risk of disability and help in the determination of preclinical sarcopenia.

This study aims to investigate the mechanisms by which income level affects skeletal muscle health among rural older residents in China, and to introduce dietary knowledge as a mediating variable. By defining a clear research objective, we can gain insight into the influencing factors of skeletal muscle health among rural older residents, especially the relationship between income level and dietary knowledge. Therefore, this study focuses on the following questions: do higher income levels and increased dietary knowledge reduce the prevalence of sarcopenia? By what mechanism do they exert this effect? Compared with the existing literature, the possible academic contributions of this study include: In addition to quantitatively analyzing the effects of income level and dietary knowledge on the population of sarcopenia patients and their food nutrient intake, it is more important to clarify the mechanism of the influence of income on the incidence of sarcopenia in rural older people by constructing theoretical models and empirical tests, which is of great significance for enhancing the dietary nutritional system of the population and promoting the implementation of the strategy of Healthy China.

## 2 Research framework and methods

### 2.1 Research framework

**Figure 1:**
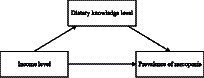
Theoretical model.

There is a hypothesis that improving the income level of older residents in rural areas can have an impact on the skeletal muscle health status of rural older people. This is because increasing income may lead to an increase in dietary knowledge, which in turn can contribute to a lower prevalence of sarcopenia among this population. On the basis of existing literature and theory, this paper constructs a framework diagram for analyzing the mediating effect of dietary knowledge level in the relationship between income and the prevalence of sarcopenia in rural older adults (Figure 1). Three variables are involved in this study: income level, dietary knowledge, and prevalence of sarcopenia among rural older adults.

Older adults with higher incomes may have easier access to more and better foods, including foods high in protein, vitamins, and minerals. This may contribute to maintaining muscle mass and reducing the risk of sarcopenia. Since it has been documented that higher-income people are in better health and use more healthcare services (30), research hypothesis 1 is proposed:

*H1*: The higher the income level of rural older adults, the lower the prevalence of sarcopenia.

Education and health are two critical forms of human capital, and it is widely established in economics that education can produce health gains. It has been shown that the most important factor influencing the improvement of dietary quality of US residents is the increase in the level of education rather than the level of income (31) and that an increase in the level of education can lead to healthier dietary behaviors, which in turn promotes better health (32). Therefore, research hypothesis 2 is proposed:

*H2*: The higher the income level of rural older adults, the higher the level of dietary knowledge.

Further, as individuals possess a higher level of dietary knowledge, they are more likely to make food choices that promote muscle retention and growth, thereby reducing the risk of sarcopenia. Based on this understanding, we propose research hypothesis 3:

*H3*: There exists a negative correlation between the level of dietary knowledge and the prevalence of sarcopenia among rural older individuals.

Taking into consideration that relatively younger older adults are typically still within the working age range and are more vulnerable to socio-economic factors (33) such as occupation, level of education, and socio-economic status.

If the income level is comparatively low, it may affect their nutritional intake, health status, and access to healthcare behaviors. Therefore, research hypothesis 4 is proposed:

*H4*: The relationship between income level and prevalence of sarcopenia varies across age groups.

### 2.2 Model setting

In this study, we constructed a mediation effect model of “income—dietary knowledge—prevalence of sarcopenia among rural older person,” and analyzed the impact of income level on sarcopenia among rural older residents, using dietary knowledge of rural older residents over 65 years old as the mediator variable. The mediating effect model of “dietary knowledge-prevalence of sarcopenia” was used to analyze the effect of income level on sarcopenia among rural older people aged over 65, with dietary knowledge as the mediating variable. The basic idea is to first analyze the effect of income level on the prevalence of sarcopenia among rural older people to obtain the total effect of income on the prevalence of sarcopenia among rural older people, then analyze the direct effect of income level on dietary knowledge among rural older people, and finally analyze the effects of income level and dietary knowledge on the health of older people’s skeleton muscles to obtain the mediating effect of dietary knowledge.

The baseline model is constructed as follows:


(1) $$ASMI_{i}=\alpha_{0}+\alpha_{1}\mathrm{incom}e_{i}{+}_{r}C+\mu_{1i}$$

(2) $$DK_{i}=\beta_{0}+\beta_{1}\mathrm{incom}e_{i}{+}_{r}\;C+\mu_{2i}$$

(3) $$ASMI_{i}=\chi_{0}+\chi_{1}\mathrm{incom}e_{i}+\chi_{2}DK_{i}{+}_{r}C+\mu_{3i}$$

$ASMI_{i}$ is individual skeletal muscle health and is a continuous variable, $\mathrm{incom}e_{i}$ is the individual income of the observer, $DK_{i}$ is a mediating variable indicating the level of dietary knowledge of the observer, C is a control variable, and $\mu_{i}$ is an error term. $\alpha_{1}$ in Eq. (1) denotes the total effect of income of rural older residents on skeletal muscle health index, in Eq. (2), $\beta_{1}$ denotes the effect of income of rural older people on the level of dietary knowledge, and in Eq. (3) $\chi_{1}$ denotes the direct effect of the level of income of rural older people on the index of skeletal muscle health, and $\chi_{2}$ denotes the mediating variable the level of dietary knowledge on skeletal muscle health effect.

Substituting Eq. (2) into Eq. (3) yields:

(4) $$ASMI_{i}=\left(\chi_{0}+\chi_{2}\beta_{0}\right)+\left(\chi_{1}+\chi_{2}\beta_{1}\right)\mathrm{incom}e_{i}{+}_{r}\;C+\mu$$

In Eq. (4) $\left(\chi_{0}+\chi_{2}\beta_{0}\right)$ denotes the total effect of income on the skeletal muscle health index of the rural older residents, 𝜒1 denotes the direct effect of income on the skeletal muscle health index of the rural older residents, and 𝜒2𝛽1 denote the indirect effect of the level of dietary knowledge. 𝜒2𝛽1/ 𝛼1 is the mediating effect of dietary knowledge.

## 3 Data sources and variable selection

### 3.1 Data sources

Data for this study come from the China Health and Nutrition Survey (CHNS). This survey is jointly conducted by the Carolina Population Center at the University of North Carolina at Chapel Hill and the National Institute of Nutrition and Health (NINH) of the Chinese Center for Disease Control and Prevention (CDC), with the aim of understanding how broader social and economic changes in China are affecting nutritional and health-related outcomes among Chinese residents. The CHNS is a continuous survey conducted annually among approximately 4,000 urban and rural households in nine provinces in China (i.e., Guangxi, Guizhou, Henan, Heilongjiang, Hubei, Hunan, Jiangsu, Liaoning, and Shandong; in 2011, the three cities of Beijing, Chongqing, and Shanghai were included). The CHNS is a continuous survey of approximately 4,400 households in urban and rural areas in nine provinces in China (i.e., Guangxi, Guizhou, Henan, Heilongjiang, Hubei, Hunan, Jiangsu, Liaoning, and Shandong; in 2011, the cities of Beijing, Chongqing, and Shanghai were included). The nine provinces include northern and southern regions, developed eastern coastal areas, and poor remote areas, which vary widely in terms of geography, economic development, public resources, and health indicators. The sample was selected through a multi-stage randomized clustering strategy and can therefore be taken as representative of the Chinese population.

Older people’s own basal metabolism decreases, their body functions in all aspects decline, and physical activity is insufficient, which is more likely to lead to sarcopenia. At the same time, the living environment of the rural older residents is relatively closed, lack of external information and communication, it is difficult to come into contact with new dietary knowledge levels. Therefore, the focus group in this paper is the rural older residents over 65 years old. In this study, the data from the three surveys in 2006, 2009, and 2011 were selected because the survey only began to ask the interviewed individuals for dietary knowledge information in 2004, but the differences between the question options in 2004 and the subsequent surveys led to systematic differences between the dietary knowledge in 2004 and the data from the subsequent three surveys, and the data on dietary nutrition of the population in 2015 have not yet been opened.

### 3.2 Variable selection

#### 3.2.1 Explained variables

The main clinical manifestations of sarcopenia are muscle weakness, which reduces the mobility of the rural older residents, causing difficulties in completing daily movements such as walking, sitting and standing, climbing and lifting heavy objects, and even leading to balance disorders, difficulty in standing, and a high susceptibility to falls. Many methods can be used to assess skeletal muscle mass, skeletal muscle force, and skeletal muscle function. Specific assessment criteria are based on AWGS recommendations and include muscle strength, extremity skeletal muscle mass (ASM), and physical performance.

Extremity Skeletal Muscle Mass: The AWGS 2019 cut-off values for low muscle mass in the diagnosis of sarcopenia are as follows: <7.0 kg/m^2^ for men and < 5.4 kg/m^2^ for women by DXA, and < 7.0 kg/m^2^ for men and < 5.7 kg/m^2^ for women by BIA. Skeletal muscle mass in the Chinese population was physically measured using the Wen et al. formula to estimate it (34). The calculation formula is as follows:


$$\begin{array}{l} ASM=0.193\times \mathrm{weight}\;\left(\mathrm{kg}\right)+0.107\times \mathrm{height}\;\left(\mathrm{cm}\right)\\ \;-4.157\times \mathrm{gender}-0.037\times \mathrm{age}\;\left(\mathrm{years}\right)-2.631 \end{array}$$

Gender is set to 1 if male and 0 otherwise. Several studies have shown that ASM calculated using this formula agrees well with dual-energy X-ray absorptiometry (DXA). In 2014, the Asian Working Group on Sarcopenia (AWGS) developed cut-offs for the measurement of sarcopenia in Asian populations: Muscle mass is measured as ASMI, and the cut-offs for males and females when applying bio refractor measurement (BIA) are the cut-off values for males and females were 7.0 kg/m^2^ and 5.7 kg/m^2^, respectively, when measured by bioelectrical impedance measurement (BIA).

#### 3.2.2 Explanatory variables

##### 3.2.2.1 Measurement of dietary knowledge

In order to measure dietary knowledge, three composite indicators were established to measure the level of residents’ dietary knowledge based on the questions in the dietary knowledge table of the CHNS questionnaire. The dietary knowledge section of the CHNS questionnaire includes 9 questions (Table 1). The answers to these questions are not always correct, therefore, the judgment of each question is given by this paper with reference to the criteria of Zhang Zongli et al. (35) as follows, as shown in the table.

**Table 1:** Question design and statistic index of dietary knowledge.

Question	Presentation of the problem	Judgment
1	Eating a diet with plenty of fruits and vegetables is good for health.	T
2	Eating more sugar is good for health.	F
3	Eating different kinds of food is good for health.	T
4	Eating foods high in fat is good for health.	F
5	Eating a diet with lots of staples is not good for health.	T
6	Eating a lot of meat every day is good for health	F
7	Eating less meat and animal fat at meals is good for health.	T
8	Drinking milk and eating dairy products are good for health.	T
9	Beans and soy products are good for health.	T

For each question in the dietary knowledge questionnaire, respondents were asked to give their answers from five options: strongly disagree, disagree, neutral, agree, and strongly agree. In this paper, we refer to Zhang Zongli et al.’s method of treatment and assign the five indicators of strongly disagree, disagree, neutral, agree, and strongly agree to scores of 1, 2, 3, 4, and 5, with higher scores indicating higher levels of dietary knowledge. Not all of the answers to the nine questions were correct, and higher scores for questions 2, 4, and 6 in the questionnaire indicated lower levels of dietary knowledge. Therefore, the scores for these three questions were re-directed so that higher scores indicated higher levels of dietary knowledge for all questions. The selection of indicators was based on Zhou et al.’s study, in which the samples’ answers to the dietary knowledge questions were judged as “correct” and “incorrect,” with one point for a correct answer to the question and zero points for the other answers (36). The scores of the nine questions for each sample were summed up as a composite indicator of their dietary knowledge endowment.

##### 3.2.2.2 Income level

Total household income is calculated from nine sources of income including business, farming, fishing, and gardening. *Per capita* household income is obtained by dividing total household income by household size. A logarithmic term for income was added based on the possible non-linear relationship between income and skeletal muscle health.

#### 3.2.3 Control variables

Much of the current literature suggests that personal and family characteristics can have an impact on the level of skeletal muscle health in older adults. In order to make the results of the analysis more accurate, personal and family characteristics and other variables were selected as control variables in this paper. Individual characteristic variables include height, weight, age, education level, and family size.

### 3.3 Descriptive statistical analysis

Descriptive statistical analysis is provided in Table 2.

**Table 2:** Definition of main variables and descriptive statistical analysis.

Variable name	Statistical analysis	Obs	Mean	SD	Min	Median	Max
Skeletal muscle mass	Calculated	1,496	0.172	0.377	0.000	0.000	1.000
Log *per capita* household income	Calculated	1,496	9.155	0.908	4.804	9.230	13.017
Age	Actual age in the year of interview	1,496	70.756	4.974	65.000	69.000	91.000
Education level	0 = None; 1 = Primary school graduate; 2 = Junior high school graduate; 3 = High school graduate; 4 = College; 5 = Bachelor’s degree	1,496	1.106	1.330	0.000	1.000	5.000
Family size	Based on the CHNS database	1,496	3.229	1.872	1.000	2.000	13.000
Working time	Based on the CHNS database	1,496	2.586	3.117	0.000	1.000	18.000
Dietary knowledge	Calculated	1,496	6.316	2.068	0.000	7.000	9.000

### 3.4 Correlation and covariance test

Before the regression, the test of Pearson correlation coefficient matrix was carried out first, and the test results are shown in Table 3, which indicated that the core explanatory variable of *per capita* household income and the prevalence of sarcopenia had a significant negative correlation, which was consistent with the expected hypotheses, and that the control variables, such as age and level of education, had a significant correlation with the dependent variable at least at the significance level of 1%, but the results are for reference only, taking into account the fact that the correlation coefficient matrix only measured the relationship between the two variables. However, considering that the correlation coefficient matrix only measures the relationship between the two variables and does not exclude the interference of control variables and potential variables, the results are for reference only, and the specific relationship needs to be determined by further regression analysis. In addition, by determining whether the absolute value of the correlation coefficient between the explanatory variables is greater than 0.9, we can also preliminarily rule out the possibility of covariate covariance.

**Table 3:** Correlation coefficient matrix.

Variable	Prevalence of sarcopenia	Log *per capita* household income	Age	Education level	Family size	Working time	Dietary knowledge
Prevalence of sarcopenia	1.000						
Log *per capita* household income	−0.110***	1.000					
Age	0.135***	0.082***	1.000				
Education level	−0.040	0.278***	−0.053**	1.000			
Family size	0.093***	−0.267***	−0.084***	−0.062**	1.000		
Working time	0.102***	−0.233***	−0.258***	−0.272***	0.065**	1.000	
Dietary knowledge	−0.082***	0.202***	−0.011	0.254***	−0.045*	−0.174***	1.000

*** *p* < 0.01, ** *p* < 0.05, * *p* < 0.1.

The dietary knowledge questionnaire in the CHNS data is set up with multiple questions to measure the level of dietary knowledge of individuals by asking their views. While multiple indicators can provide rich information for the study from different perspectives and reflect the level of dietary knowledge of the residents in a more comprehensive way, they also increase the complexity and difficulty of the analysis, and there may be a certain degree of correlation between different indicators, and the multiple covariances will lead to large errors in the estimation results. In order to avoid covariance in the data, a multicollinearity test is needed, which is generally used to detect whether there is multicollinearity through the variance inflation factor VIF: it is the ratio of the variance when there is multicollinearity between the explanatory variables to the variance when there is no multicollinearity. The larger the inverse of the tolerance VIF, the more serious the covariance. The empirical judgment method shows that: when 0 < VIF < 10, there is no multicollinearity; when 10 ≤ VIF < 100, there is strong multicollinearity; when VIF ≥ 100, there is severe multicollinearity. Table 4 shows the results of the multicollinearity test of the model, and it can be seen that the VIF value of each variable is less than 10, thus overall, the indicators selected in this paper do not have covariance.

**Table 4:** Covariance test.

Variable	VIF	1/VIF
Working time	1.201	0.833
Log *per capita* household income	1.196	0.836
Education level	1.163	0.860
Age	1.098	0.910
Family size	1.081	0.925
Mean VIF	1.148	0.873

## 4 Results

### 4.1 Baseline regression analysis of the effect of income on the prevalence of sarcopenia among rural older people

In this study, we first conducted a baseline regression of income and prevalence of sarcopenia among rural older people using the Probit model to clarify the relationship between income and prevalence of sarcopenia among rural older residents. Table 5 reports the regression results of income on the prevalence of sarcopenia among rural older residents. The regression results in columns (1), and (2) show that income is significant at the 1% level where the regression coefficient of the logarithm of *per capita* household income is negative indicating that the prevalence of sarcopenia decreases as the income level increases. Based on this result, hypothesis 1 of this study was tested.

**Table 5:** Baseline regression results.

Variable name	(1)	(2)
	Prevalence of sarcopenia	Prevalence of sarcopenia
Log *per capita* household income	−0.178*** (−4.32)	−0.141*** (−3.11)
Age		0.055*** (6.75)
Education level		0.036 (1.14)
Family size		0.065*** (3.05)
Working time		0.066*** (5.07)
Constant	0.669* (1.78)	−4.016*** (−5.52)
Observations	1,496	1,496
R^2^	0.0130	0.0586

*, **, **** indicate significant at the 10, 5, and 1% levels, respectively; standard deviations are in parentheses. Prevalence of sarcopenia (1) is without control variables; (2) is with control variables.

Income level has a significant negative impact on the prevalence of sarcopenia among rural older residents, and there is a potential endogeneity issue when exploring the relationship between income level and skeletal muscle health among rural older people in terms of *per capita* household income. Specifically, on the one hand, personal income affects BMI, which in turn affects skeletal muscle health; at the same time, BMI also affects *per capita* household income by influencing skeletal muscle health, so it can be inferred that skeletal muscle health also affects the level of *per capita* household income; on the other hand, some omitted variables are related to both *per capita* household income and skeletal muscle health. Potential endogeneity problems can lead to biased estimates of the coefficients of the independent variables.

The instrumental Variable Method is widely used to solve the endogeneity problem, “Instrumental Variable Probit” (IV Probit) and(the two-step Method) are academically recognized as two effective methods for testing the endogeneity of the Probit model (37), the bi-directional relationship between skeletal muscle health and income in this study can lead to endogeneity problems in the model, the potential endogeneity between income and the prevalence of sarcopenia may affect the stability of the conclusions, and the Probit model was used in this study, so the use of the instrumental variable Probit method can effectively address the endogeneity of income. Taking into account the availability of data, this study will refer to Tian and Yu’s selection of the instrumental variable for income in the CHNS (38), and use the variable “how many televisions the household has that can be watched” as the instrumental variable for income, and use the instrumental variable estimation method of 2SLS for the comparative analyses. The reason is that wealth status is a potentially valid instrumental variable for household income level, and “how many TVs the household can watch” can effectively represent household wealth.

Table 6 reports the regression results, the first stage regression results show that “the number of TV sets available for viewing in the household” has a significant positive effect on “household income *per capita*,” i.e., “the number of TV sets available for viewing in the household” has a good explanatory power on the level of household income *per capita*. In other words, “the number of TV sets that can be watched at home” has good explanatory power for the level of *per capita* household income, and the variable “the number of TV sets that can be watched at home” can be used as an instrumental variable to satisfy the test of relevance, which can help to accurately identify the impact of income level on skeletal muscle health. Further endogeneity test, the Wald test indicates that *per capita* household income is an endogenous variable, which should be corrected by the instrumental variable method. In order to better address the endogeneity of the model, a weak instrumental variable test was conducted, the test results significantly rejected the original hypothesis, indicating that “how many TV sets can be watched in the household” is not a weak instrumental variable. Considering that the number of instrumental variables is equal to the number of endogenous variables, the instrumental variables are identified exactly.

**Table 6:** Instrumental variable regression of *per capita* income of older rural residents.

Variable name	Stage 1	Stage 2
	*Per capita* household income	Revised *per capita* household income
How many TVs are available for viewing in the household	0.303*** (10.60)	
Log *per capita* household income		−0.751*** (−3.61)
Age	0.008* (1.70)	0.059*** (6.79)
Education level	0.132*** (7.98)	0.132*** (2.82)
Family size	−0.157*** (−12.72)	−0.008 (−0.25)
Working time	−0.032*** (−4.12)	0.040** (2.41)
Constant	8.708*** (26.08)	1.462 (0.74)
Observations	1,496	1,496
Wald test (P > chi^2^)	0.0015	

Probit regression coefficients are shown in the tables and standard errors are in parentheses. The coefficients on per capita household income in the tables are those estimated using instrumental variables. *** *p* < 0.01, ** *p* < 0.05, * *p* < 0.1.

### 4.2 Analysis of the mechanism of action of the effect of income on the prevalence of sarcopenia in rural older people

#### 4.2.1 Mediating effects of income on the prevalence of sarcopenia in rural older people through dietary knowledge levels

In this study, the mediation effect test of nutritional intake was conducted using the mediation effect model, and the validation of the mediation effect of the variable level of dietary knowledge was carried out using the stepwise method, and the results are shown in Table 7. The first step was to verify the relationship between the independent and dependent variables, i.e., the effect test of *per capita* household income and the prevalence of sarcopenia, which was partially demonstrated in the baseline regression in the previous section, i.e., *per capita* household income was negatively correlated with the prevalence of sarcopenia at the 1% level of significance. In the second step, the dependent variable was replaced with the mediator variable to investigate whether there was a significant correlation between the independent variable and the mediator variable, and the results showed that *per capita* household income was positively correlated with dietary knowledge at the 1% significance level, and the significant relationship was established. The third step is to add the mediator variable on the basis of the first step and conduct regression again, if the mediator variable is significant, it indicates that there is a mediation effect, and the results show that the level of dietary knowledge is positively correlated with the prevalence of sarcopenia at the 1% significance level, so the mediation effect exists, and the level of dietary knowledge of the older person in rural areas mediates the role of knowledge of the level of income and the prevalence of sarcopenia.

**Table 7:** Intermediation effect.

Variable name	(1)	(2)
	Dietary knowledge	Prevalence of sarcopenia
Log *per capita* household income	0.296*** (4.69)	−0.131*** (−2.88)
Dietary knowledge		−0.034* (−1.82)
Age	−0.016 (−1.35)	0.054*** (6.70)
Education level	0.292*** (8.13)	0.047 (1.43)
Family size	0.005 (0.17)	0.065*** (3.06)
Working time	−0.068*** (−3.88)	0.063*** (4.88)
Constant	4.547*** (4.46)	−3.847*** (−5.30)
Observations	1,496	1,496
R^2^	0.092	–
Adj.R^2^	–	0.0608

*** *p* < 0.01, ** *p* < 0.05, * *p* < 0.1.

#### 4.2.2 Heterogeneity analysis of the effect of income on the prevalence of sarcopenia among rural older people through their level of dietary knowledge

Table 8 provides heterogeneity analysis.

**Table 8:** Heterogeneity analysis.

Variable name	Age 65–70	Age over 70
	Prevalence of sarcopenia	Prevalence of sarcopenia
Log *per capita* household income	−0.157** (−2.52)	−0.119* (−1.80)
Age	0.069* (1.73)	0.062*** (4.49)
Education level	0.024 (0.48)	0.052 (1.21)
Family size	0.064** (2.17)	0.057* (1.86)
Working time	0.039** (2.34)	0.100*** (4.85)
Constant	−4.694* (−1.67)	−4.841*** (−3.95)
Observations	757	739
R^2^	0.0355	0.0681

*** *p* < 0.01, ** *p* < 0.05, * *p* < 0.1.

#### 4.2.3 Robustness test of the effect of income on the prevalence of sarcopenia in rural older people through dietary knowledge levels

In order to test the robustness of the findings, we used Logit and LPM methods to estimate the intervention effect again. Firstly, the regression analysis was carried out using the Logit model, which eliminated the effect of extreme data and allowed us to study the factors influencing the prevalence of sarcopenia in rural older residents more accurately. Then the LPM model was used to test the results again, and it can be seen that under both models, the prevalence of sarcopenia in rural older people is negatively correlated with *per capita* household income the results are significant, and the results of the study are basically the same as the previous article. Therefore, by combining the above robustness results, the findings of this study have a certain degree of reliability (see Table 9).

**Table 9:** Robustness test.

Variable name	Logit	LPM
	Prevalence of sarcopenia	Prevalence of sarcopenia
Log *per capita* household income	−0.034*** (−2.87)	−0.240*** (−3.03)
Age	0.014*** (6.36)	0.097*** (6.72)
Education level	0.010 (1.30)	0.066 (1.15)
Family size	0.016*** (2.78)	0.111*** (2.96)
Working time	0.016*** (4.82)	0.116*** (5.15)
Constant	−0.618*** (−3.17)	−7.103*** (−5.49)
Observations	1,496	1,496
R^2^	0.054	0.0578

*** *p* < 0.01, ** *p* < 0.05, * *p* < 0.1.

## 5 Conclusions and implications

### 5.1 Conclusion

This study examined the mediation effect of income and dietary knowledge on the prevalence of sarcopenia among older rural residents using data from the China Health and Nutrition Survey (CHNS) in 2006, 2009, and 2011. The main conclusions are as follows:

(1) The higher the income level of rural older adults, the lower the prevalence of sarcopenia. This finding is consistent with the conclusion proposed by Zhang et al. that advanced-age individuals with cognitive decline, low income, smoking, malnutrition, and decreased exercise time are risk factors associated with age-related skeletal muscle atrophy (39).
(2) In this study, the level of dietary knowledge was used as a mediating variable to reveal the mechanism of action by which income level affects skeletal muscle health in rural older adults. The empirical results showed that the higher the income level of rural older residents, the higher the level of dietary knowledge. Meanwhile, dietary knowledge was negatively correlated with the prevalence of sarcopenia among rural older residents. Therefore, for older residents with sarcopenia, improving their dietary knowledge can effectively reduce their calorie intake and achieve the dual effects of controlling body weight and maintaining and increasing muscle mass.
(3) Dietary knowledge level played an intermediary role in income level and the prevalence rate of sarcopenia in rural older residents. This suggests that increased dietary knowledge can have an impact on income levels and the prevalence of sarcopenia. Increased knowledge of dietary nutrition and effective nutritional guidance can help older rural residents improve their dietary habits and increase their nutritional intake to improve their quality of life and skeletal muscle health.
(4) The relationship between income level and the prevalence of sarcopenia among rural older adults varies by age. There is heterogeneity in the effect of income growth on the prevalence of sarcopenia among older rural residents, and this effect is more pronounced for the relatively younger older age groups.

The main contribution of this study is that it focuses on the mechanism of the influence of income on the prevalence of sarcopenia in rural older adults from the perspective of dietary knowledge level, which makes up for the current lack of research on the intrinsic mechanism of the influence of income on skeletal muscle health indices in rural older residents. However, due to data limitations, this paper fails to analyze the impact of dietary knowledge acquisition on the skeletal muscle health of rural older residents. This study will provide a reference for the governmental authorities to formulate policies to improve the dietary knowledge of the population.

### 5.2 Enlightenment

WHO proposes that health assessment indicators for the older residents focus on the ability to live independently, i.e., whether they are able to live on their own, rather than just death and illness (40). The main purpose of this paper is to investigate the influencing factors of sarcopenia among rural older people based on their income level and dietary knowledge and to take timely and effective interventions to minimize the incidence of sarcopenia and prevent the reduction of muscle strength from affecting the ability of older people to take care of themselves, so as to improve the quality of life of the older people and to prolong their lifespan.

Based on the conclusions of the above studies, the following policy recommendations are put forward to reduce the prevalence of sarcopenia in China, accelerate the construction of a healthy China with the people as the center, and implement the strategy of a healthy China: Firstly, increasing the income of farmers is still an important means to improve the dietary health level of the rural residents and to reduce the prevalence of sarcopenia in rural residents. Vigorously supporting the lower income groups in rural areas to achieve sustainable growth of low income, but also to respond to the national policy requirements of “precise poverty alleviation,” and at the same time to improve the level of dietary knowledge of rural residents to improve their skeletal muscle health. Secondly, government departments, nutrition, and health organizations, educational institutions, and other organizations and institutions should formulate more concise dietary guidelines, widely disseminate dietary knowledge, actively promote a rational diet, and focus on giving dietary guidance to people suffering from sarcopenia. Through regular nutritional knowledge lectures and the distribution of healthy diet brochures, the dietary knowledge of the Chinese population can be increased, and unhealthy dietary behaviors can be adjusted. Thirdly, it is paying attention to the dietary and nutritional situation of low-income groups of rural older people, promoting balanced nutrition through the distribution of food vouchers, and the strengthening of nutritional dietary publicity, so as to reduce the incidence of sarcopenia and improve the health of the population.

## Data availability statement

The original contributions presented in the study are included in the article/supplementary materials, further inquiries can be directed to the corresponding authors.

## Ethics statement

Ethical approval was not required for the studies involving humans because the data obtained by this study is not private and is anonymous and therefore does not require ethical approval. The studies were conducted in accordance with the local legislation and institutional requirements. The participants provided their written informed consent to participate in this study.

## Author contributions

XZ: Formal analysis, Writing – original draft. GW: Conceptualization, Writing – review & editing. JM: Visualization, Writing – review & editing. HB: Supervision, Writing – review & editing.
